# “To die is better than to tell”: reasons for and against disclosure of chronic hepatitis B status in Ghana

**DOI:** 10.1186/s12889-020-08811-5

**Published:** 2020-05-12

**Authors:** Charles Ampong Adjei, Sarah E. Stutterheim, Florence Naab, Robert A. C. Ruiter

**Affiliations:** 1 Department of Work and Social Psychology Maastricht University, Maastricht, The Netherlands; 2 Department of Community Health Nursing, University of Ghana, Accra, Ghana; 3 Department of Maternal and Child Health, University of Ghana, Accra, Ghana

**Keywords:** Hepatitis B, Disclosure, Non-disclosure, Ghana

## Abstract

**Background:**

People with a condition subject to stigmatisation, such as chronic hepatitis B, face the dilemma of whether or not to disclose their status. In Ghana, 12.3% of the adult population has the hepatitis B virus (HBV). One key strategy for breaking the cycle of hepatitis B transmission is the disclosure of hepatitis B status by people with chronic hepatitis B (PWHB). Disclosure can facilitate preventive actions to reduce hepatitis B transmission (e.g., not sharing personal items and avoiding contact with blood and body fluids). Disclosure can also motivate family members of PWHB to get tested, linked to care and clinically managed in order to reduce the progression of hepatitis B to liver cirrhosis and cancer. Given the importance of disclosure, we set out to explore reasons for and against disclosure of chronic hepatitis B status in the Greater Accra and Upper East region of Ghana.

**Methods:**

In this exploratory qualitative study, 18 participants (10 from the Greater Accra region and 8 from the Upper East region) were recruited for semi-structured interviews. Interviews were recorded and transcribed verbatim. Data were then processed using QSR Nvivo version 10.0 and analysed for themes.

**Results:**

Participants were selective disclosers, disclosing in some contexts and not in others. Reasons for non-disclosure of chronic hepatitis B status were: 1) fear of stigmatisation and 2) previous negative experiences with disclosure. Reasons for disclosure were: 1) wanting close contacts to get tested or vaccinated, 2) trusting the disclosure target(s), and 3) needing social and/or financial support.

**Conclusions:**

Our findings highlight various reasons for and against disclosure of chronic hepatitis B status in Ghana. Because anticipated, observed, and experienced stigma were important motivations for non-disclosure of chronic hepatitis B status, we recommend the development and implementation of theory and evidence-based stigma reduction interventions that are culturally appropriate, and that prioritize the participation of target populations. We also recommend the provision of counselling and support services that assist PWHB in their disclosure decision-making processes.

## Background

People with a condition subject to stigmatisation, such as chronic hepatitis B, are often faced with the dilemma of whether or not to disclose their status. This decision is dependent on various factors. People are more likely to disclose when they perceive the overall benefits of disclosure to outweigh the costs [1–3]. According to the disclosure decision-making theory, three processes occur before disclosure ensues, namely information assessment, receiver assessment, and disclosure efficacy [4, 5]. Information assessment is an evaluation of the risks and rewards disclosing. Receiver assessment weighs the anticipated reaction of the disclosure target. Lastly, disclosure efficacy reflects the discloser’s level of confidence to discuss the condition [4, 5].

In the case of chronic hepatitis B, disclosure can serve as a primary prevention intervention [6–8]. This is particularly crucial in high endemic countries such as Ghana, where transmission of hepatitis B predominantly occurs within families as a result of exposure from mother to child [9]. In these regions, disclosure of chronic hepatitis B status can facilitate preventive action and the avoidance of actions that lead to hepatitis B transmission, such as sharing personal items (e.g., razors, toothbrushes) and contact with blood and body fluids (e.g., saliva, menstrual fluid, and vaginal secretions) [9]. Hepatitis B status disclosure can also lead undiagnosed family members to get tested and linked to care, which reduces the progression of the disease to liver cirrhosis and cancer. Disclosure can also offer PWHB opportunities to receive support [8], which can lead to better treatment adherence and emotional well-being [10, 11].

However, disclosure of chronic hepatitis B status can also have potentially negative consequences. It can lead to rejection [12], shame [13], embarrassment [14], avoidance [15], stigma, and discrimination [6, 7, 16, 17]. For example, in a study conducted by Wallace et al. [18] in Australia with PWHB, participants reported divorce and denial of sex as negative consequences of disclosure. Similarly, in Rafique and colleagues’ [16] study on the experiences of stigma among PWHB in Pakistan, about half of the participants reported avoidance of sexual relations by their spouse, and societal and family discrimination, as negative consequences of disclosure.

There have been a number of studies that have looked at the complexities associated with disclosure of chronic hepatitis B status in countries such as Australia, Vietnam, and China [7, 18, 19], but our understanding of what motivates or impedes disclosure in the sub-Saharan African context, where hepatitis B prevalence rates are above 8% [20], is very limited. To our knowledge, there is only one other study, conducted in Zambia, that has investigated disclosure of chronic hepatitis B status in an African context [6]. We therefore, in this study, set out to explore reasons for and against hepatitis B status disclosure among people with chronic hepatitis B in Ghana.

### Hepatitis B testing and care in Ghana

The prevalence rate of hepatitis B in Ghana is 12.3% [21]. Ghana has a national viral hepatitis control programme. In fact, a national guideline for prevention, care, and treatment of viral hepatitis was developed in 2015, and relaunched in July of 2019. However, the full implementation of the national guideline is yet to be funded by the government. As a result, voluntary testing for hepatitis B currently requires out-of-pocket payment costing between $4 and $6 in public and private health facilities. Additionally, vaccination of new-born children whose mothers have chronic hepatitis B within 24 hours following birth is not universally implemented due to a lack of funding. Further, the costs of hepatitis B clinical monitoring (e.g., viral load monitoring and liver scans) and treatment are not covered by the national health insurance scheme but, rather, carried by affected individuals and/or their families. Only blood donors, pregnant women, and, in some instances, professionals who engage in exposure-prone procedures, such as healthcare providers, are screened for hepatitis B without a fee. In light of these challenges, disclosure of hepatitis B status by PWHB is likely the most feasible avenue for primary (i.e., avoidance of actions that can lead to transmission) and secondary prevention (i.e., screening of close contacts) of hepatitis B in Ghana.

## Methods

### Study design and setting

We used an exploratory qualitative design [22–24]. Participants were recruited from two hospitals in Ghana: a teaching hospital in the Greater Accra region and a regional hospital in the Upper East region. In the Greater Accra region, patients with chronic hepatitis B access care through the liver clinic where specialists provide services. In the Upper East region, patients with chronic hepatitis B are attended to by general practitioners at the outpatient department. This study is part of a larger study on psycho-social challenges with chronic hepatitis B in Ghana [15, 25]. Here, we report on the findings pertaining to reasons for and against disclosure of chronic hepatitis B status. We previously published a paper on hepatitis B related stigma in Ghana that focused on the determinants and manifestations of hepatitis B stigma. The paper can be accessed

at: https://bmjopen.bmj.com/content/bmjopen/9/6/e025503.full.pdf." Ethics approval was provided by the Korle-Bu Institutional Review Board (Protocol No. KBTH-IRB 00092/2016).

### Participants and recruitment

In total, 18 PWHB participated in the study. Participants were included if they were (1) Ghanaian born, (2) aged 18 years and above, and (3) had tested hepatitis B surface antigen (HBsAg) positive at least six (6) months (i.e, an indication of chronic infection per WHO criteria) prior to recruitment. We excluded one participant who was in the terminal stage of hepatitis B as he had insufficient energy to do an interview. We used purposive sampling to recruit participants who met the inclusion criteria [26, 27].

Posters with details of the study, including the purpose of the study, information about confidentiality, the voluntary nature of the study, and the procedure for registration were advertised in the selected health facilities. This yielded six participants. Nurses also recruited PWHB in the hospitals, yielding another 12 participants. In total, 18 participants consented and participated in the study. Of the 18 participants, 10 were recruited via the teaching hospital in the Greater Accra region and the remaining 8 were recruited through the tertiary hospital in the Upper East region. Two PWHB in the Greater Accra region who were contacted by the nurses refused to participate in the study. One cited time constraints and the other person declined to provide reasons for refusal. Participants were contacted two days before the interview by telephone to remind them of the time and venue for the interview.

### Data collection

Data were collected between February and November 2017. At the start of the interviews, participants were provided again with information about the purpose of the study. Following informed consent, the interview, which ranged from 45-60 minutes, commenced. The interviews were conducted face-to-face mostly in the participant’s homes, and mainly under trees. The first author (CAA), who is a male PhD candidate with a qualitative research background, conducted the interviews in English. Throughout the interview, a probing technique was employed to garner as much relevant information as possible [28]. Field notes were also taken during and after the interviews. Also, after the interview, demographic characteristics such as age, gender, occupation, marital status, year of hepatitis B diagnosis, and how the participants came to know their hepatitis B status were noted. Data saturation was reached after the 14^th^ interview [22, 29, 30]. However, an additional four interviews were conducted to ensure that saturation was indeed reached.

### Research instrument

A semi-structured interview guide was specifically developed for this study based on the hepatitis B literature [11, 15] and was also reviewed extensively by a qualitative research methods expert (SS). The guide was then piloted with two PWHB. The topics explored during the interviews, that are reported upon here, include: (1) whether or not participants had informed someone about their chronic hepatitis B status, (2) if they had not, reasons for not disclosing, and (3) if they had, reasons for disclosing. The core interview questions are presented as Additional file 1.

### Data processing and analyses

Data were processed with QSR Nvivo version 10.0. The first author (CAA) listened to the audio recordings and transcribed verbatim. We then conducted an inductive thematic analysis [31]. The first transcribed data were coded by two of the authors (CAA and SS), followed by discussions on the individual codes, categories, and themes generated. Codes and categories that differed were discussed until consensus was reached. Throughout the process of analysis, categories and main themes, and any changes made to them, were documented. Preliminary findings were checked with two representatives of the study population to establish if the findings agreed with their views.

## Results

### Demographic characteristics

Overall, 18 PWHB from the Greater Accra (*n*=10) and Upper East (*n*=8) region of Ghana participated. Ages ranged from 21 to 57 years (*M*=35.33, *SD*=8.84), and participants had lived with chronic hepatitis B between 1 and 7 years (*M*=5.06, *SD*=1.86). Testing for hepatitis B was self-initiated (*n*=6), physician-initiated (*n*=1), part of outreach screening services (*n*=5), or part of prenatal care (*n*=6). A summary of participant’s demographic characteristic is presented in Table 1.

**Table 1:** Demographic characteristics of the participants

Participant number	Marital status	Year of Diagnosis	How participants were diagnosed
PWHB 1	Single	2014	Self- initiated
PWHB 2	Married	2011	Hospital protocol for pregnant women
PWHB 3	Single	2013	Hospital protocol for pregnant women
PWHB 4	Single	2016	Physician initiated
PWHB 5	Married	2016	Hospital protocol for pregnant women
PWHB 6	Married	2012	Hospital protocol for pregnant women
PWHB 7	Married	2015	Self-initiated
PWHB 8	Married	2012	Outreach screening programme
PWHB 9	Single	2016	Outreach screening programme
PWHB 10	Married	2011	Outreach programme
PWHB 11	Married	2011	Outreach programme
PWHB 12	Single	2015	Self-initiated
PWHB 13	Married	2011	Hospital protocol for pregnant women
PWHB 14	Married	2014	Outreach programme
PWHB 15	Married	2012	Self-initiated
PWHB 16	Single	2011	Self-initiated
PWHB 17	Married	2013	Hospital protocol for pregnant women
PWHB 18	Single	2015	Self-initiated

### Reasons for non-disclosure of chronic hepatitis B status

Participants were selective in their disclosure; they disclosed in some contexts and not in others. Among participants who chose not to disclose their hepatitis B status in certain contexts, reasons for non-disclosure included fear of stigmatisation and previous negative experiences with disclosure.

#### Fear of stigmatisation

The majority of the participants believed that negative perceptions about hepatitis B held by the general public lead to negative reactions toward PWHB. These reactions included avoidance, gossip, and possible job loss. In an effort to avoid these stigmatising reactions and keep social relations intact, several participants hid their hepatitis B status from others. This was particularly the case in the context of friendships.

*“Are you not in this country? Haven’t you seen that most people are very careful with those with hepatitis B? If you want people to look at you some way and treat you like someone with a deadly disease then you tell them of your status. As for me, I don’t want such treatment from my friends so I won’t mention to any of them. I know that once they get to know they will change the way they flow with me. Hanging out and eating together, chilling and so on will all end.”* (PWHB 2, Greater Accra Region)

Non-disclosure was frequently triggered by anticipated stigma. This was further illustrated by a participant who feared that disclosure would lead to problems in her romantic relationship.

*“My result* (HBsAg positive test result) *currently is still in my head. I have not told him* (boyfriend)*. He is also a medical person and that scares me more. There are times I have even wanted a break up because I feel when he gets to know it will be a negative point.”* (PWHB 1, Greater Accra Region)

Participants also reported fearing gossip if they disclose. The narrative of some participants clearly showed that they were not prepared to openly discuss their hepatitis B status with others because they were convinced that they would then become the subject of gossip among their peers and close contacts. A participant who was diagnosed with chronic hepatitis B in school shared her view as follows:

*“I didn't inform anyone, not even the person who sent me to the hospital knew about it. I told the doctor not to disclose it to any other person because I didn’t want to be discussed by my friends in school.”* (PWHB 4, Greater Accra Region)

Another participant also said,

*“You know, letting people know is not the answer to it now. They will only see me as someone with a deadly disease and begin to discuss me like the way they do to AIDS patients.”* (PWHB 15, Upper East Region)

Another form of stigmatisation feared by some of our participants was loss of employment. This was a concern for the two participants working in the food industry as caterers. These participants indicated that making their status known to their employers would lead to job loss. Although there is no policy in Ghana that limits PWHB involvement in the food industry, one participant indicated that people are often uncomfortable eating food prepared by PWHB because they fear they will get hepatitis B. Also, food industry employers are often unwilling to employ PWHB, as indicated by another participant.

**“***Because of how people talk about hepatitis B, I know when my madam* [employer] *gets to know that I am positive, she will sack me because we deal with food.”* (PWHB 3, Greater Accra Region)

*“I am the one who prepares my director’s lunch at the office. I have done this for more than five years and I have never mentioned to my boss that I am hepatitis B positive. Even though I know how to handle myself so I don’t give him the disease, I am sure he won’t be happy to eat my food once I tell him.”* (PWHB 16, Greater Accra Region)

Clearly, fear of stigmatisation was a prominent reason for not disclosing hepatitis B status, as exemplified by the following comment from a PWHB living in the Upper East region:

*“I will remain quiet about my hepatitis B status because I don’t want people to give me special treatment. You know, I live by the saying, to die is better than to tell and be ashamed. I am not ready to tell anyone now. Maybe in future but not now.”* (PWHB 13, Upper East Region)

#### Previous negative experiences with disclosure

Another motivation for non-disclosure was previous negative experiences with disclosure. One negative experience reported was third party disclosure. Participants indicated choosing not to disclose because they had previously disclosed and had their confidentiality violated by their confidant.

*“The only one I trusted and informed was my uncle, and he spread it to the family members, and they were afraid of me.”* (PWHB 3, Greater Accra Region)

Another previous negative experience was disclosure targets feeling uncomfortable and uneasy around the PWHB after disclosure. This was considered to be rooted in disclosure targets’ fear of infection from the PWHB. One woman narrated how her romantic relationship was threatened after informing her partner of her hepatitis B status.

*“I experienced a couple of changes in my boyfriend’s behaviour towards me. As soon as I told him that I have hepatitis B, he started giving me some little gaps. I can see that he is not very comfortable around me. Even though we are still dating, his love had become a bit cold, unlike when I had not informed him about my status.”* (PWHB 9, Upper East Region)

In sum, the reasons against disclosure were fear of stigmatisation and previous negative experiences with disclosure.

### Disclosure of chronic hepatitis B status

In addition to the providing reasons for chronic hepatitis B status non-disclosure, participants also shared reasons for disclosing their hepatitis B status to selected individuals. These were wanting close contacts to get tested and vaccinated, trusting the disclosure target, and needing social and/or financial support.

#### Wanting close contacts to get tested and vaccinated

The most frequently cited reason for disclosure was wanting to have close contacts get tested and vaccinated. This was rooted in participants’ belief that they could be a source of hepatitis B infection to their close relations and that disclosure could reduce their close contacts’ potential exposure to the virus. However, even participants with transmission concerns were, in spite of their desire to protect others, still cautious about who they tell. The disclosure targets often included only immediate family members (i.e., parent(s) and sibling(s) and sexual partner(s), and this was reported to be necessitated by the fact that these people were household members who could reasonably be at risk for hepatitis B exposure and possible infection.

*“I informed my mum and my sisters aside from my husband. I live with them and therefore deemed it necessary to inform them so they can get tested and receive the vaccine.”* (PWHB 5, Upper East Region)

*“Because I knew I could pass the infection to my boyfriend through sex, I felt it was important to tell him so that he can prevent himself by taking the hepatitis B vaccination.”* (PWHB 16, Greater Accra Region)

#### Trusting the disclosure target(s)

Another reason for disclosure reported by our participants centred on the trust they had in their disclosure target(s). The majority of our participants said they informed only people they knew to be trustworthy enough to handle their hepatitis B status with the utmost secrecy.

*“Some people when you tell them you have hepatitis B they will broadcast it. As for my husband, I knew he would not tell others. That is why I did not inform those who are not closer to me but my husband alone.”* (PWHB 14, Greater Accra Region)

Others also disclosed their hepatitis B status because discussing personal problems with family members is considered the norm in their family.

*“In my family, we don’t keep things to ourselves, we share our success and difficult moments, so I felt it was important to open up to my mother and let her know what was going on.”* (PWHB 16, Greater Accra Region)

#### Needing social and/or financial support

Another reason for disclosure reported by participants was that they needed support relating to treatment or care. In Ghana, the costs of clinical monitoring and treatment of chronic hepatitis B are very expensive for the average Ghanaian. Unfortunately, government subsidies have yet to be extended to chronic hepatitis B treatment and care. However, in many Ghanaian societies, informal support systems are available to family members in the event of illness and bereavement. Some of our participants thus disclosed to family who could assist them financially.

“*In an event of illness, you can’t do away with family. Sometime a word from someone can encourage you to keep the hope alive. Beside, because they were my family and they take care of me, I had to let them know”* (PWHB 4, Greater Accra Region)

Others also deemed it necessary to inform those who had funded the cost of their hepatitis B test. According to one participant, her uncle demanded the hepatitis B test report he paid for, and that led to the discovery of her positive hepatitis B status.

*“I took money from my uncle to do the test and when I brought the result; he checked and realised I was hepatitis B positive”* (PWHB 3, Greater Accra Region)

In summary, the reasons for disclosure were wanting close contacts, including immediate family members and sexual partners, to get tested and vaccinated, trusting the disclosure target, and needing social and/or financial support. These are presented in Table 2.

**Table 2:** Summary of findings

Themes	Sub-Themes
1. Reasons for non-disclosure of chronic hepatitis B status	• Fear of stigmatization • Previous negative experiences with disclosure
1. Disclosure of chronic hepatitis B status	• Wanting close contacts to get tested and vaccinated. • Trusting the disclosure target (s) • Needing social and/or financial support

## Discussion

This study is one of the first to explore reasons for and against disclosure of chronic hepatitis B status in sub-Saharan Africa, and the first to do this in Ghana. Overall, our findings have shown that PWHB chose not to disclose their hepatitis B status because they 1) fear stigmatisation and 2) have had previous negative experiences with disclosure. Reasons for disclosure were: 1) wanting close contacts to get tested and vaccinated, 2) trusting the disclosure target(s), and 3) needing social and/or financial support.

Fear of stigmatisation was identified as an important reason for not disclosing chronic hepatitis B status. This concern is unsurprising given the plethora of evidence that hepatitis B is associated with stigma [32–34], also in Ghana [15]. This is primarily related to the perceived infectiousness of hepatitis B [16, 35]. The majority of participants chose not to disclose their hepatitis B status to avoid being gossiped about. In collectivistic cultures like Ghana, people speak openly about their health challenges with close contacts in order to, for example, receive emotional, spiritual, and physical support. Unfortunately, the proliferation of erroneous information about chronic hepatitis B within the social space, particularly regarding the means of transmission [11], seems to inhibit the fulfilment of this cultural norm. Our finding that many participants would not share their hepatitis B status with others because they feared gossip exemplifies this, and supports findings from Franklin and colleagues [6], who in their study of hepatitis B disclosure and screening in Zambia, found that some participants kept their hepatitis B status from others because they feared becoming the subject of discussion. Similar concerns were also established by Sriphanlop et al. [36] in their study of factors relating to hepatitis B screening among Africans with hepatitis B in New York. They found that participants were uncomfortable having their status known in their community because they feared gossip.

Furthermore, fear of losing employment was identified as an important reason for non- disclosure of chronic hepatitis B status. This was reported by two of our participants working in the food industry. This concern may be linked to incorrect knowledge about hepatitis B transmission routes among these participants, which may have led them to believe that hepatitis B can be transmitted through the sharing of food and utensils [15]. Alternatively, our participants were responding to the belief that PWHB should not be allowed to work in restaurants [19].

Our findings further showed that, in addition to fear of stigmatisation in the form of gossip, or job loss, PWHB were deterred from disclosing because they had witnessed the stigmatisation of others. Our participants felt that the reactions towards people living with HIV (PLHIV) are not very different from reactions to PWHB. They therefore felt the need to keep their hepatitis B status to themselves. Others ostensibly mentioned that they had drawn lessons from witnessing the stigma experiences of both PWHB and PLHIV,and that this was their reason for non-disclosure. This is likely the result of hepatitis B often being associated with HIV, which is highly stigmatised in Ghana [37]. In fact, in Ghana, hepatitis B is often misconstrued to be more dangerous than HIV [11], and, in light of the already extensive stigmatisation of PLHIV, it is likely that the associations made between hepatitis B and HIV strengthened our participants’ commitment not to disclose their chronic hepatitis B status. The perception that hepatitis B is more dangerous than HIV is driven by a lack of knowledge, often resulting from the provision of inaccurate hepatitis B information by herbal practitioners on various media platforms in Ghana [38]. One common message used by herbal practitioners to attract customers to purchase their products is that hepatitis B is more dangerous than HIV.

Our findings also established a number of reasons for disclosure. One was wanting to protect families from hepatitis B. Participants outlined that disclosure was necessary so that their family members could get tested and vaccinated. This was because many participants perceived themselves to be a possible source of hepatitis B infection to their family members. This belief has also been observed in other locales, such as the USA [32], Malaysia [39], and Iran [40]. This finding is also particularly encouraging in light of the World Health Organisation’s (WHO) recommendation to increase hepatitis B testing [41].

Furthermore, our findings showed that, in light of fears of stigmatisation, most participants engaged in selective disclosure as a way of limiting the possible negative consequences of disclosure. In congruence with other studies, immediate family members and sexual partners appeared to be the most frequent disclosure targets [6, 14, 19]. In Franklin and colleagues [6] study, spouses and first-degree relatives of PWHB were also the most common disclosure targets. Also, in a study conduct by Huang et al. [19] in China, the majority of participants disclosed their hepatitis B status more frequently to siblings, spouses, and children than to their friends [15]. In our view, selective disclosure was preferred either because 1) participants feared negative consequences of hepatitis B disclosure including stigmatisation, rejection, embarrassment, and discrimination [14, 19, 42] or 2) because participants were aware of the high prevalence of hepatitis B within families, as a result of mother-to-child transmission [9]..

Another reason for disclosure was needing social and/or financial support. This is in line with a broad literature demonstrating that disclosure is often a prerequisite for support [1, 43–45]. In our study, the type of support PWHB benefited from following disclosure was social support or financial aid. No previous literature on hepatitis B disclosure has demonstrated this. However, Stutterheim et al. [46] did established that PLHIV who disclosed their status to others benefited from social support, and that this was associated with reduced psychological distress. What was interesting in our study is that the individuals that financed participants’ tests demanded information regarding the outcome of those tests which compelled a couple of our participants to disclose their hepatitis B status. This can be viewed in terms of the disclosure decision-making theory that recognises the role of rewards versus risks as important determinants of disclosure [4, 5].

Our findings have some important implications. In terms of theory, our study makes an important contribution to a very limited literature on hepatitis B status disclosure in Africa. We contend that more studies in this area are necessary, particularly in light of hepatitis B prevalence rates in the region. In Ghana specifically, we recommend following up on this qualitative study with a quantatitive study, to better establish the frequency of these reasons for disclosure and non-disclosure, and their impact, while also considering important contextual variables such as gender, location, and age. Given the role of stigma in Ghanaian PWHB’s disclosure decisions, we also recommend invetigating perceptions of hepatitis B related stigma among PWHB using a validated hepatitis B stigma scale.

Our study also has important practical implications. The finding that fear of stigmatisation discourages PWHB from disclosing points to the need for stigma reduction interventions. We specifically recommend the development and implementation of theory and evidence-based stigma reduction interventions that are culturally appropriate, and that prioritize the participation of target populations and stakeholders from the outset. Also, given that disclosure contributes to the prevention of hepatitis B transmission within families, we believe that increasing the health literacy of PWHB and their immediate family members could effectively contribute to reducing further transmission of the hepatitis B. Additionally, we recommend the provision of counselling and support services that aid in disclosure decision-making processes of PWHB and that provide PWHB with a safe place to weigh the advantages and disadvantages of disclosing or not disclosing their hepatitis B status.

The findings of this study should be viewed in light of some limitations. The first is a potential selection bias. In our study, substantially more women than men participated. This may have impacted the findings such that the views of women with hepatitis B were better represented than the views of men with hepatitis B. However, we believe that not much variation would have been observed had more men been included. Also, the exclusion of PWHB who were in the terminal stage of the disease could potentially be considered a limitation. Perhaps, their inclusion would have provided a different view to that expressed by our participants because these people are likely to manifest visible signs of hepatitis B. Furthermore, bias may have resulted from our choice to recruit participants from hospital settings. PWHB recruited via hospitals may be more likely than other PWHB to recognise the importance of managing hepatitis B and may also have more resources to seek formal care. As a result, the views expressed by our participants might not be in line with those of PWHB who do not access formal care in hospitals.

## Conclusions

This study has outlined reasons for and against chronic hepatitis status disclosure in the Greater Accra and Upper East region of Ghana. Reasons for non-disclosure of chronic hepatitis B status reported were: 1) fear of stigmatisation, and 2) previous negative experiences with disclosure. Reasons for disclosure were: 1) wanting close contacts to get tested or vaccinated, 2) trusting the disclosure target(s), and 3) needing social and/or financial support. Because anticipated, observed, and experienced stigma were important motivations for non-disclosure of chronic hepatitis B status, we recommend the development and implementation of theory and evidence-based stigma reduction interventions that are culturally appropriate, and that prioritize the participation of target populations. We also recommend the provision of counselling and support services that assist PWHB in their disclosure decision-making processes.

## Supplementary information

**Additional file 1:.** Interview guide

## Data Availability

All data generated or analysed during this study are included in this published article and its supplementary information files.

## Abbreviations

PWHB: People with Hepatitis B
PLHIV: People living with HIV
