# Supplementary material for: “To die is better than to tell”: reasons for and against disclosure of chronic hepatitis B status in Ghana
Source: BMC Public Health. 2020 May 12;20:663. doi: 10.1186/s12889-020-08811-5 (PMC7216649; doi:10.1186/s12889-020-08811-5)
Supplement: Supplementary file 1 — Additional file 1:. Interview guide [file 12889_2020_8811_MOESM1_ESM.docx]

**Supplementary File 1: Interview guide**

1. Kindly tell me about yourself (e.g., age, marital status, how you got to know your hepatitis B status, and year of diagnosis)
2. Have you informed someone about your HBV status?

If yes:

- Can you please share with me the person you informed about your HBV status?
- Why did you decide to inform them?
- How did you go about telling that person?
- How did the person react at the time?
- Were there other reactions later? If any, please describe.

If no:

- Why did you decide not to inform anyone?
- What were your reasons for not telling anyone?
